# Supplementary material for: Structural Genomic Variation and Its Potential Role in Deer Speciation
Source: Mol Ecol. 2026 May 7;35:e70365. doi: 10.1111/mec.70365 (PMC13150800; doi:10.1111/mec.70365)
Supplement: Supplementary file 1 — Figure S1: Short‐read structural variant workflow. Species are abbreviated as follows: MD = Mule deer, WTD = White tailed deer. Figure S2: Long‐read structural variant workflow. Species are abbreviated as follows: MD = Mule deer, WTD = White tailed deer. Table S1: Percentage of homozygous structural variants (SVs) detected in the short read and long read analyses (Figures S1 and S2) across all mapping strategies (MD2MD, MD2WTD, WTD2MD, WTD2WTD). For each SV type (deletion, duplication, insertion, inversion, translocation), the proportion of homozygous events was calculated as the number of homozygous SVs divided by the total SVs detected in that scenario. Table S2: Mule and white‐tailed deer genes found within fixed species–specific structural variants and present in 100% of samples. Gene name from the white‐tailed deer annotation with Orthogroup ID. Type of structural variant (SV). Significant p‐values calculated by BUSTED (Murrell et al. 2015) and Bonferroni‐Holm corrected are denoted by * on the Orthogroup ID. Citations for putative male reproductive and olfactory links of impacted genes are provided. Table S3: Motif density in mule deer (MD) and white‐tailed deer (WTD) genomic regions (genic regions masked). Comparisons include gene bodies, ±50 kb gene flanking regions, 10,000 random SVs (±50 kb), and 27 fixed SVs (±50 kb). Final column reports the standard deviation of the average motif density values (Avg Motifs/bp). [file MEC-35-e70365-s001.docx]

**Supplementary Material**

**Figure S1.** Short-read structural variant workflow. Species are abbreviated as follows: MD = Mule deer, WTD = White tailed deer.

**
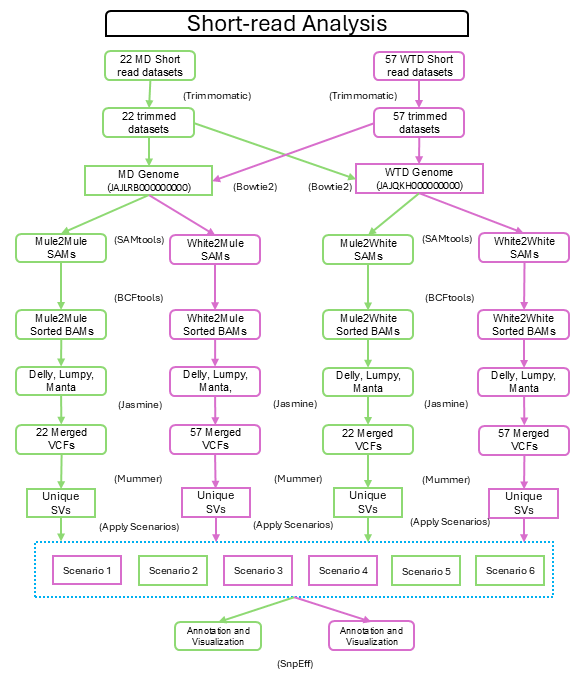
**

**Figure S2.** Long-read structural variant workflow. Species are abbreviated as follows: MD = Mule deer, WTD = White tailed deer.

**
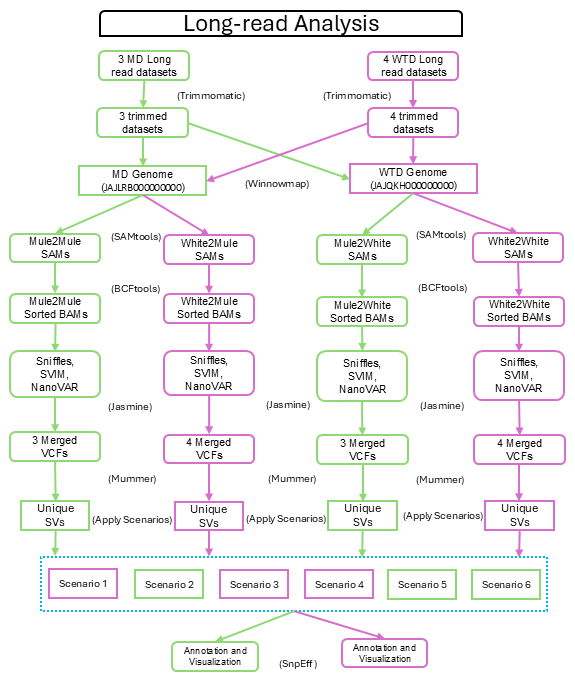
**

**Table S1.** Percentage of homozygous structural variants (SVs) detected in the short read and long read analyses (Figure S1 & S2) across all mapping strategies (MD2MD, MD2WTD, WTD2MD, WTD2WTD). For each SV type (deletion, duplication, insertion, inversion, translocation), the proportion of homozygous events was calculated as the number of homozygous SVs divided by the total SVs detected in that scenario.

|  | MD2MD | | MD2WTD | | WTD2MD | | WTD2WTD |
| --- | --- | --- | --- | --- | --- | --- | --- |
|  | **Short read SV analysis** | | | | | | |
| Deletion | 74.93 | 93.49 | | 77.66 | | 66.21 | |
| Duplication | 6.17 | 3.28 | | 1.42 | | 1 | |
| Insertion | 77.01 | 78.73 | | 68.84 | | 63.86 | |
| Inversion | 24.94 | 44.22 | | 24.65 | | 20.92 | |
| Translocation | 4.87 | 2.92 | | 3.69 | | 3.52 | |
|  | **Long read SV analysis** | | | | | | |
| Deletion | 39.39 | 68.3 | | 54.36 | | 41.39 | |
| Duplication | 28.65 | 27.07 | | 35.4 | | 29.18 | |
| Insertion | 43.17 | 63.59 | | 48.55 | | 39.62 | |
| Inversion | 25.93 | 58.17 | | 37.5 | | 37.56 | |
| Translocation | 1.32 | 7.65 | | 18.44 | | 15.98 | |

**Table S2.** Mule and white-tailed deer genes found within fixed species–specific structural variants and present in 100% of samples. Gene name from the white-tailed deer annotation with Orthogroup ID. Type of structural variant (SV). Significant P-values calculated by BUSTED (Murrell et all, 2015) and Bonferroni-Holm corrected are denoted by * on the Orthogroup ID. Citations for putative male reproductive and olfactory links of impacted genes are provided.

| **Orthogroup ID** | **Gene Name** | **SV-type** | **SV-size (≈ bp)** | **Potential Speciation link** |
| --- | --- | --- | --- | --- |
| OG0016858 | MERTK | Translocation | 483 bp | Male reproduction - (Shi et al, 2023) |
| OG0016300* | CD70 | Translocation | 340 bp | - |
| OG0016712 | LOC110146842 | Translocation | 307 bp | Olfactory receptor – (NCBI, 110146842) |
| OG0017397 | KCNK1 | Translocation | 775 bp | Male reproduction and olfactory  - (Delgado-Bermúdez et al, 2025; Yu et al, 2024) |
| OG0017682* | LRRC38 | Translocation | 287 bp | - |
| OG0017020 | OLFML2A | Translocation | 1 kbp | - |
| OG0015447* | COL19A1 | Duplication | 86 kbp | - |
| OG0016445 | FABP6 | Deletion | 1.3 Mbp | - |
| OG0017723 | TASL | Translocation | 1 kbp | - |
| OG0014825 | IGFBP3 | Translocation | 1 kbp | Male reproduction - (Fu et al, 2021) |
| OG0016518 | PAPPA2 | Translocation | 1 kbp | Male reproduction - (Zamil et al, 2024) |
| OG0014866 | CREB3L2 | Duplication | 40 kbp | - |
| OG0016712 | LOC110146842 | Inversion | 9 Mbp | Olfactory receptor – (NCBI, 110146842) |
| OG0015201 | TLCD1 | Translocation | 733 bp | - |
| OG0016445 | FABP6 | Translocation | 573 bp | - |
| OG0015643 | TAS1R2 | Inversion | 3 Mbp | Male reproduction and olfactory - (Mosinger et al, 2013; Zhao et al, 2010) |
| OG0014825 | IGFBP3 | Deletion | 559 bp | Male reproduction - (Fu et al, 2021) |
| OG0015470 | TUBE1 | Translocation | 1356 bp | Male reproduction - (Stathatos et al, 2024) |
| OG0015643 | TAS1R2 | Translocation | 418 bp | Male reproduction and olfactory - (Mosinger et al, 2013; Zhao et al, 2010) |
| OG0014866 | CREB3L2 | Duplication | 40 kbp | - |
| OG0015681 | SLC16A8 | Translocation | 732 bp | - |
| OG0017560 | EDNRB | Insertion | 1 kbp | Olfactory - (Bryche et al, 2020) |
| OG0016858 | MERTK | Translocation | 1 kbp | Male reproduction - (Shi et al, 2023) |
| OG0014916 | LOC110148892 | Deletion | 1 kbp | - |
| OG0015201 | TLCD1 | Translocation | 733 bp | - |
| OG0016518 | PAPPA2 | Deletion | 5 kbp | Male reproduction - (Zamil et al, 2024) |
| OG0013553 | RXRG | Deletion | 1 kbp | Male reproduction - (Wang et al, 2020) |

**Table S3.** Motif density in mule deer (MD) and white-tailed deer (WTD) genomic regions (genic regions masked). Comparisons include gene bodies, ±50 kb gene flanking regions, 10,000 random SVs (±50 kb), and 27 fixed SVs (±50 kb). Final column reports the standard deviation of the average motif density values (Avg Motifs/bp).

| **Data** | **Mean # Motifs** | **Avg Motifs / bp** | **Standard Deviation** |
| --- | --- | --- | --- |
| Genome (intergenic) | 481.06 | 0.01099 | 0.01978 |
| Genic regions +/- 50Kb | 296.1475 | 0.00594 | 0.00540 |
| Random SV +/- 50Kb | 122.37 | 0.00246 | 0.00211 |
| Fixed SVs +/- 50Kb | 35.99 | 0.00078 | 0.00024 |

**References**

Bryche, B., A. Saint-Albin, C. Le Poupon Schlegel, C. Baly, P. Congar, and N. Meunier. 2020. Endothelin increases the proliferation of rat olfactory mucosa cells. Neural Regeneration Research 15:352–360.

Delgado-Bermúdez, A., M. Yeste, S. Bonet, and E. Pinart. 2025. Physiological role of potassium channels in mammalian germ cell differentiation, maturation, and capacitation. Andrology 13:184–201.

Fu, L., K. C. J. Yuen, A. N. Tint, A. R. Hoffman, A. T. Bongso, and K. O. Lee. 2021. Association of decreased sperm motility and increased seminal plasma IGF-I, IGF-II, IGFBP-2, and PSA levels in infertile men. Endocrine 74:698–706.

Mosinger, B., K. M. Redding, M. R. Parker, V. Yevshayeva, K. K. Yee, K. Dyomina, Y. Li, and R. F. Margolskee. 2013. Genetic loss or pharmacological blockade of testes-expressed taste genes causes male sterility. Proceedings of the National Academy of Sciences of the United States of America 110:12319–12324.

Murrell, B., S. Weaver, M. D. Smith, J. O. Wertheim, S. Murrell, A. Aylward, K. Eren, T. Pollner, D. P. Martin, D. M. Smith, K. Scheffler, and S. L. Kosakovsky Pond. 2015. Gene-wide identification of episodic selection. *Molecular Biology and Evolution* 32:1365–1371.

Stathatos, G. G., D. J. Merriner, A. E. O’Connor, et al. 2024. Epsilon tubulin is an essential determinant of microtubule-based structures in male germ cells. EMBO Reports 25:2722–2742.

Wang, G. S., A. Liang, Y. B. Dai, X. L. Wu, and F. Sun. 2020. Expression and localization of retinoid receptors in the testis of normal and infertile men. Molecular Reproduction and Development 87:978–985.

Yu, Y., P. Liao, and R. Jiang. 2024. Ion channels in odor information processing of neural circuits of the vertebrate olfactory bulb. International Journal of Molecular Sciences 25:13259.

Zamil, Z. S., M. M. Abduulraheem, G. M. Khaleel, and S. K. Ibraheem. 2024. Assessing the potential of mainstream pregnancy-associated plasma protein A (PAPP-A) level in men as a biomarker for fertility: A review. Journal of Basrah Researches (Sciences) 50:77–85.

Zhao, H., Y. Zhou, C. M. Pinto, P. Charles-Dominique, J. Galindo-González, S. Zhang, and J. Zhang. 2010. Evolution of the sweet taste receptor gene Tas1r2 in bats. Molecular Biology and Evolution 27:2642–2650.
